# Association between Weight Loss and Food Form in Older Individuals Residing in Long-Term Care Facilities: 1-Year Multicenter Longitudinal Study

**DOI:** 10.3390/ijerph182010776

**Published:** 2021-10-14

**Authors:** Akemi Endo, Yutaka Watanabe, Takae Matsushita, Kazutaka Okada, Yuki Ohara, Masanori Iwasaki, Kayoko Ito, Junko Nakajima, Yasuyuki Iwasa, Masataka Itoda, Rikimaru Sasaki, Yasuhiro Nishi, Junichi Furuya, Yoshihiko Watanabe, George Umemoto, Masako Kishima, Hirohiko Hirano, Yuji Sato, Mitsuyoshi Yoshida, Yutaka Yamazaki

**Affiliations:** 1 Gerodontology, Department of Oral Health Science, Faculty of Dental Medicine, Hokkaido University, Hokkaido 060-8586, Japan; st-endou@biz.nifty.jp (A.E.); tkea-224@den.hokudai.ac.jp (T.M.); kz@den.hokudai.ac.jp (K.O.); yutaka8@den.hokudai.ac.jp (Y.Y.); 2 Tokyo Metropolitan Institute of Gerontology, Tokyo 173-0015, Japan; yohara@tmig.or.jp (Y.O.); iwasaki@tmig.or.jp (M.I.); h-hiro@gd5.so-net.ne.jp (H.H.); 3 Oral Rehabilitation, Niigata University Medical and Dental Hospital, Niigata 951-8520, Japan; k-ito@dent.niigata-u.ac.jp; 4 Department of Oral Medicine and Hospital Dentistry, Tokyo Dental College, Chiba 272-8513, Japan; jun.k.nakajima@gmail.com; 5 Department of Dentistry, Haradoi Hospital, Fukuoka 813-8588, Japan; y_iwasa@haradoi-hospital.com; 6 Department of Oral Rehabilitation, Osaka Dental University Hospital, Osaka 573-1144, Japan; m.itoda89@gmail.com; 7 Rehabilitation Clinic for Speech and Swallowing Disorders, The Nippon Dental University Hospital, Tokyo 102-8158, Japan; rikimaru@tky.ndu.ac.jp; 8 Department of Oral and Maxillofacial Prosthodontics, Kagoshima University Graduate School of Medical and Dental Sciences, Kagoshima 890-8544, Japan; shar@dent.kagoshima-u.ac.jp; 9 Department of Geriatric Dentistry, Showa University School of Dentistry, Tokyo 145-8515, Japan; furuya-j@dent.showa-u.ac.jp (J.F.); sato-@dent.showa-u.ac.jp (Y.S.); 10 Department of Healthcare Management, Tohoku Fukushi University, Miyagi 981-8522, Japan; yoshiw@tfu-mail.tfu.ac.jp; 11 Swallowing Disorders Center, Fukuoka University Hospital, Fukuoka 814-0180, Japan; george@fukuoka-u.ac.jp; 12 Wakakusa-Tatsuma Rehabilitation Hospital, Osaka 574-0012, Japan; tdbqd500@yahoo.co.jp; 13 Department of Advanced Prosthodontics, Hiroshima University Graduate School of Biomedical & Health Sciences, Hiroshima 734-8553, Japan; mitsu@hiroshima-u.ac.jp

**Keywords:** food form, long-term care facility, nursing care, oral function, swallowing function, weight loss

## Abstract

Changing the food form for older adults requiring nursing care from a regular to dysphagia diet is thought to impact their nutritional status. We assessed the association between changes in food form and weight loss over 1 year in older adults. Older adults residing in long-term care facilities in Japan (*n* = 455) who participated in the baseline (2018) and follow-up (2019) surveys were divided into two groups (regular diet, *n* = 284; dysphagia diet, *n* = 171). The regular diet group was further divided into the weight loss (*n* = 80; weight loss ≥5% over 1 year) and weight maintenance (*n* = 204; weight loss <5%) groups. After 1 year, the Barthel Index significantly decreased, and the proportion of participants who switched from a regular diet to a dysphagia diet significantly increased in the weight loss group than in the weight maintenance group. Multivariate logistic regression analysis found that Barthel index variation (odds ratio (OR): 0.97, 95% confidence interval (CI): 0.94‒0.99), change from a regular diet to a dysphagia diet (OR: 4.41, 95% CI: 1.87‒10.41), and body weight at baseline (OR = 1.06, 95% CI: 1.01‒1.11) were significantly associated with weight loss. Our results suggest that maintaining the food form inhibits weight loss and improves health outcomes in older adults.

## 1. Introduction

Using data from a study involving 10,298 residents of 191 nursing homes in 13 countries that participated in the “Nutrition Day in nursing homes” project (2007–2012), Wirth et al. reported that a low body mass index (BMI, <20 kg/m^2^) and ≥5 kg weight loss are important factors associated with 6-month mortality [1]. This suggests that maintaining the body weight in long-term care facility residents is vital for preserving their health.

Japan has the highest rate of increase in the number of elderly people worldwide [2,3], accompanied by a surge in the number of older adults requiring nursing care and residents of long-term care facilities requiring continuous advanced care [4]. The efficacy of standard interventions for improving nutritional status is reduced in most older adults who require nursing care due to reduced cognitive function or eating and swallowing dysfunction [5,6].

Malnutrition resulting from a reduced eating and swallowing function in older adults requiring nursing care has been found to worsen the level of care and have a major impact on survival [7]. A change from a regular to a dysphagia diet has been reported to decrease both nutritional quality and quality of life (QOL) [5,8,9,10,11]. Reduced eating and swallowing functions increase the risk of accidents, such as asphyxia and pulmonary aspiration. Studies have shown that long-term care facilities prioritize the prevention of such accidents over maintenance of food form, and, therefore, often switch residents’ diet to a dysphagia diet [12].

The change to a dysphagia diet is thought to negatively impact the nutritional status of older adults requiring nursing care. However, the number of studies on this subject is small. We hypothesized that changing a regular diet to a dysphagia diet is related to weight loss in older adults requiring nursing care. If it is proven that maintaining food form is an effective way to maintain the nutritional status of older adults requiring nursing care, long-term care facilities may place greater emphasis on maintaining and improving the food form and improving the eating and swallowing function of residents. In this study, we used weight loss as an indicator of nutritional status and aimed to clarify the association between weight loss and food form changes in older adults requiring nursing care in long-term care facilities in Japan.

## 2. Materials and Methods

### 2.1. Study Population

**Figure 1:**
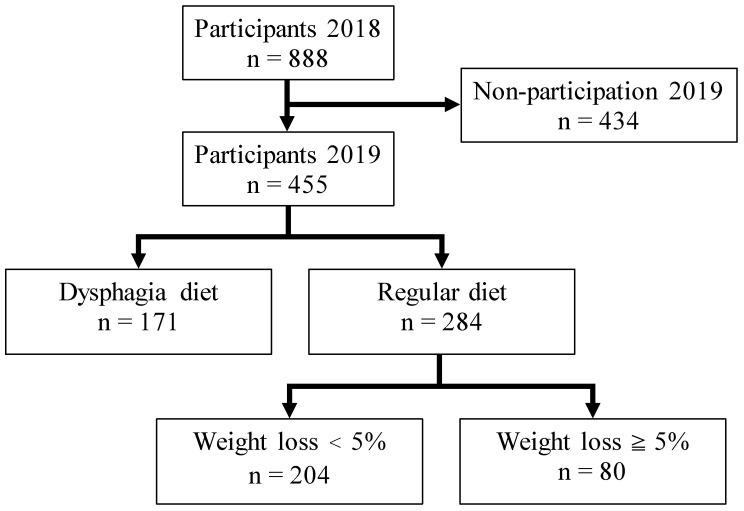
Flowchart of study participants.

Before conducting the survey, 30 members of the Japanese Society of Gerodontology Study Working Group received training on evaluation standards of the study to ensure consistency in the study methods. The trained members explained what the study was about to the directors and staff members of the long-term care facilities to which they were affiliated. Thirty-seven facilities from 17 regions of Japan agreed to participate. In September 2018, these facilities provided a written explanation of the study to all residents and their families. Written consent was obtained from 888 residents and their families. A baseline survey was conducted between October 2018 and February 2019. In September 2019, requests for an additional survey were sent to the 37 facilities that had participated previously. Of these, 25 provided consent for participation in the next part of the study. This time, written informed consent was obtained from 455 residents who had participated in the previous year’s survey. The same survey questionnaire was administered at baseline and during the 1-year follow-up (Figure 1).

### 2.2. Survey Items

Before the study started, members of the study working group trained all facility nurses and registered dietitians on how to assess the survey items, to ensure consistent assessment standards. Survey forms were then distributed to the residents’ attending nurses who filled in basic information; the Barthel index (BI), as an assessment of activities of daily living (ADL) [13]; and the clinical dementia rating (CDR) [14], as an assessment of cognitive function.

### 2.3. Survey Contents in the Questionnaire

#### 2.3.1. Basic Information

Participants’ age, sex, height, weight, BI, CDR, and medical history (pneumonia—including aspiration pneumonia, cerebrovascular disease, diabetes, and depression) were copied from the care chart.

#### 2.3.2. Total Energy Intake and Food Form

Each facility’s registered dietitian calculated the average daily total oral energy intake (kcal/day) for each resident in the previous week, with reference to the amount of food offered and uneaten. Regarding the food form, a diet corresponding to the 2013 Japanese dysphagia diet classification codes of the Japanese Society of Dysphagia Rehabilitation was considered a dysphagia diet [15,16]. Food forms not requiring any other processing or special preparation beyond the normal or that used processing or special preparation as standard (e.g., chopping or thickening with starch) were considered a regular diet.

### 2.4. Oral Survey

Fifty dentists and dental hygienists (3–10 per facility), who were trained for consistent assessment standards, visited the long-term care facilities to evaluate the residents’ oral health.

#### Evaluation of Oral Condition

Participants’ swallowing function was evaluated using a questionnaire for dysphagia screening developed by Ohkuma et al. [17]. This questionnaire consists of 15 items reflecting pneumonia history, nutritional status, oral, pharyngeal, and esophageal function, and epiglottis function. Each question is answered on a three-point scale of “A: severe symptoms,” “B: mild symptoms,” or “C: no symptoms.” As in previous studies, we rated participants as “having dysphagia” if the answer to any of the 15 items was A, “having signs of dysphagia” if the answer to any item was B, and “non-dysphagic” if all answers were C [18,19].

To determine the remaining teeth, the number of teeth within the oral cavity was counted, excluding any residual roots with destroyed crowns or teeth with severe periodontitis. The sum of the remaining teeth and dental prostheses (false teeth, dental bridges, and implants) was used as the number of functional teeth. Participants with no remaining teeth were considered edentulous. Participants who used false teeth while eating were considered prosthesis users.

### 2.5. Statistical Analysis

The participants were first classified into the dysphagia diet group and regular diet group. The regular diet group was then divided into a group with ≥5% weight loss 1 year after the baseline survey (weight loss group) and a group with <5% weight loss (weight maintenance group) based on a threshold from previous studies on weight loss and mortality in older adults [20,21]. Each item of the baseline survey was compared between the diet groups and between the weight subgroups. Changes in each survey item from baseline to follow-up (1 year later) were compared between these groups. For each two-group comparison, categorical variables were analyzed using the chi-square test, and continuous variables were analyzed using the Mann-Whitney U test for variables with a normal data distribution. Binomial logistic regression analysis was performed, and odds ratios were calculated (95% confidence interval) for the regular diet group with or without ≥5% weight loss at 1 year after the baseline survey as the dependent variable. Age, sex, weight at baseline, medical history [22], and factors associated with weight loss (BI [22], CDR [22], total energy intake [23], dysphagia [24], functional teeth [25], and food form change within 1 year) were selected as independent variables. Baseline data were subtracted from the 1 year follow-up data for BI, total energy intake, and functional teeth (i.e., negative values represented deterioration of BI, reduced total energy intake, and fewer functional teeth). Our study involved participants from multiple facilities. Participants were nested in those facilities; therefore, we performed multilevel analysis to check the necessity to control for correlations between participants within the same facility, prior to the logistic regression analysis.

All statistical analyses were performed using SPSS Statistics 26 (IBM, Armonk, NY, USA). *p* < 0.05 indicated statistical significance.

## 3. Results

In total, 455 residents participated in the baseline and 1 year follow-up surveys (85 men, 370 women; mean age: 86.5 ± 7.8 years).

Baseline weight, BI, and total energy intake, expressed as median (interquartile range (IQR)), for the total study participants were 45.2 (40.1‒51.9) kg, 30.0 (10.0‒50.0) points, and 1254.0 (1108.3‒1400.0) kcal/day, respectively. There were 403 (89.7%) participants with cognitive function decline, with a score of at least 1 on the CDR. Regarding the oral condition, 183 (40.2%) participants were classified as having a possibility of dysphagia. The average number of remaining teeth was 5.0 (0.0‒16.0), while that of the functional teeth was 26.0 (13.0‒28.0). In total, 155 (34.3%) participants were edentulous, and 243 (53.8%) were prosthesis users.

The dysphagia and regular diet groups included 171 (37.6%) and 284 (62.4%) participants, respectively. At baseline, participants in the regular diet group were younger, had a higher body weight and BI, a lower rate of cognitive function decline and swallowing dysfunction, more remaining and functional teeth, a lower rate of edentulousness and prosthesis use, and significantly fewer individuals with a history of pneumonia, than those in the dysphagia diet group (Table 1).

Of the 284 participants in the regular diet group, 80 (28.2%) constituted the weight loss group while 204 (71.8%) constituted the weight maintenance group. There were no significant differences in any survey item between these two groups at baseline (Table 2).

Comparison of changes in BI, CDR, total energy intake, number of functional teeth, and percentage of participants on a dysphagia diet between baseline and 1-year follow-up revealed that BI had decreased significantly in the weight loss group than in the weight maintenance group (Table 3).

The likelihood ratio test comparing the multilevel and single-level models did not show significant results, indicating no-random effect for facilities. Therefore, we selected a single-level (non-nested) model. Binomial logistic regression analysis of the presence or absence of weight loss ≥5% at 1 year after the baseline survey revealed significant associations between weight loss ≥5% and BI variation (OR: 0.97, 95% CI: 0.95‒0.99; *p* = 0.009), change from a regular diet to a dysphagia diet (OR: 3.02, 95% CI: 1.41‒6.46; *p* = 0.004), and body weight at baseline (OR = 1.04, 95% CI: 1.00‒1.08; *p* = 0.031) (Table 4).

## 4. Discussion

The results of this study confirmed a significant association between ≥5% weight loss over a 1-year period and a change from a regular to a dysphagia diet among older adults residing in a long-term care facility and requiring nursing care.

The study participants were older adults requiring advanced nursing care, and many of them had reduced cognitive function. These factors make it difficult to implement interventions intended to improve these residents’ nutritional status [5,6]. Older adults have a high risk of malnutrition, which has a negative impact on their overall health due to deterioration of their nutritional status [26]. It is, therefore, essential to identify ways to maintain and improve their nutritional status and to perform simple and effective interventions. Our results indicated the area for intervention: maintenance of food form. We believe that food form (i.e., eating and swallowing function) is a factor that can be maintained and even improved with appropriate interventions [27,28].

Some studies reported that approximately 26–67% long-term care insurance facility residents consume dysphagia diets to prevent pulmonary aspiration [9,12]. However, these diets are associated with problems, such as unappealing appearance and taste, low nutritional value, and risk of dehydration [5,8,9,10,11]. Our study findings might also explain why older adults requiring nursing care and who are at risk of pulmonary aspiration or asphyxia should only be put on a dysphagia diet when it is necessary.

Intervention studies are necessary in assessing the impact of changes of food form on weight loss. However, since intentionally switching patients from a regular diet to a dysphagia diet has the potential to reduce the patients’ appetite and negatively affect nutritional status, we conducted an observational study. The facility sampling was biased because the study only included facilities to which members of the Japanese Society of Gerodontology were affiliated. However, age, BMI, BI, and CDR of our study participants were similar to those of participants of other studies conducted in Japan and other countries [29,30,31].

A previous study revealed that an increased mortality rate in individuals with ≥5% weight loss over a 6-month period was caused primarily by disease, social factors, psychiatric factors, or aging [20,21]. Based on this finding, we divided the study participants (regular diet group) into a group with ≥5% weight loss and that with <5% weight loss over 1 year. We identified the predictors of weight loss using the baseline results as the independent variable; however, we ultimately chose the change over 1 year for each item to explore interventions that could inhibit weight loss.

There were significant differences at baseline in age, weight, BI, CDR, dysphagia screening rating, number of remaining and functional teeth, edentulousness, and prosthesis use, and history of pneumonia between the regular and dysphagia diet groups. These results demonstrated that participants’ food forms were generally appropriate. Simultaneously, no significant differences were found in the percentage of participants with weight loss over a year, total energy intake, or history of stroke. Regarding the percentage of participants with weight loss, the mean baseline weight of the dysphagia diet group was 5.4 kg lower than that of the regular diet group, suggesting that they were probably less likely to lose weight. For total energy intake, a difference in the dietitians’ nutritional calculations and amount of food provided would have been expected because bodyweight differed significantly; however, no significant difference was found. This may be because some participants on a regular diet had difficulties in consuming the total amount of required energy. However, there were no significant differences in any of the survey items between the weight maintenance and weight loss groups (Table 2), including total energy intake and dysphagia screening rating (an indicator of swallowing function). Thus, we believe that the inconsistency between baseline eating and swallowing function and food form is unlikely to have affected our primary results.

There were significant differences in BI changes and the percentage of participants with a change from a regular to a dysphagia diet; however, there was no significant difference in the other items (Table 3). This may be because few participants had lost their functional teeth (loss of remaining teeth or discontinuation of prosthesis use) during the observation period. Regarding the baseline swallowing function evaluation using the dysphagia screening rating, <30% of the participants had issues with swallowing. Issues with swallowing function may have become more difficult to notice when the patients’ diet was switched to a dysphagia diet, as many of the items on the scale were evaluated by observation during eating. For the CDR, 84.6% of the participants scored ≥1, and this may be because there were few participants whose CDR scores worsened. Although the change in total energy intake was not significant, this is probably because the extent of change was small.

An association between reduced total energy intake and weight loss was not found in the multivariate analysis, likely because the reduction in total calorie intake in the weight loss group was only approximately 23.5 kcal/day. However, our survey only included the weekly average total calorie intake at baseline and after 1 year. It is possible that during the observation period, eating a regular diet became difficult due to the decline in the eating and swallowing function, resulting in a marked temporary decrease in energy intake that affected weight loss. However, we were unable to collect such data in the present study. We did not investigate snacks. Participants who changed from a regular diet to a dysphagia diet may have had a restricted snack intake. This is an issue for future research. We need to consider snacks in future studies.

This study had some limitations. First, this study focused on food form; however, the participants’ food form was not determined through precise testing by an expert in eating and swallowing function. Thus, the regular diet group may have included some participants who could have been on a dysphagia diet, while the dysphagia diet group may have included some who could have been on a regular diet. Participants for whom the eating and swallowing function were inconsistent with the food form used may have experienced deterioration in their nutritional status and subsequent weight loss. Furthermore, we did not consider at what point during the observation period the food form was changed. The appropriateness of the food form and the timing of the food form was changed may have impacted our results. Second, in this study, 434 of the 888 participants in the 2018 baseline survey did not participate in the 2019 survey. Therefore, selection bias may have occurred. Third, in addition to the items examined in this study, the contribution of the main factors that dictate food form changes and the possibility that a deteriorating health status affects weight loss should be considered. However, we were not able to investigate all the factors. This study involved residents of long-term care insurance facilities in Japan. Long-term care insurance facilities in Japan provide uniform services under the long-term care insurance of the national social insurance system. Therefore, the influence of the care received, living environment, and socioeconomic factors on diet form or weight loss is expected to be small. We did not consider the development of new diseases, deterioration of comorbid disorders, or dental treatments affecting oral consumption during the observation period. This is a major limitation of this study.

## 5. Conclusions

A decline in ADL and change of food form from a regular to a dysphagia diet were associated with weight loss among residents of long-term care insurance facilities. This research demonstrated that changing a regular diet to a dysphagia diet is related to weight loss in older adults requiring nursing care.

## Figures and Tables

**Table 1:** Characteristics of study participants and comparison between the regular and dysphagia diet groups at baseline.

Variable	Overall (*n* = 455)				Dysphagia Diet (*n* = 171)				Regular Diet (*n* = 284)				*p* Value
	Mean ± SD			Median (Q1, Q3)	Mean ± SD			Median (Q1, Q3)	Mean ± SD			Median (Q1, Q3)	
	*n* (%)				*n* (%)				*n* (%)				
Age (years)	86.5	±	7.8	87.0 (82.0, 93.0)	87.5	±	7.7	88.0 (83.0, 93.0)	85.9	±	7.9	87.0 (81.0, 92.0)	0.035
Sex (female)	370		(81.3)		139		(81.3)		231		(81.3)		1.000
Weight (kg)	46.1	±	8.7	45.2 (40.1, 51.9)	42.7	±	6.8	42.7 (38.0, 47.7)	48.1	±	7.9	47.4 (41.7, 53.4)	<0.001
Weight loss ≥5%	130		(28.6)		50		(29.4)		80		(28.2)		0.830
Barthel Index	33.7	±	26.2	30.0 (10.0, 50.0)	16.5	±	19.0	10.0 (00.0, 25.0)	44.0	±	24.4	45.0 (25.0, 60.0)	<0.001
Clinical Dementia Rating													
0	9		(2.0)		2		(1.1)		7		(2.7)		<0.001
0.5	37		(8.2)		20		(2.1)		33		(12.7)		
1	93		(20.7)		33		(10.0)		74		(28.6)		
2	133		(29.6)		37		(25.3)		85		(32.8)		
3	177		(39.4)		65		(61.6)		60		(23.2)		
Total energy intake (kcal/day)	1261.0	±	215.9	1254.0 (1108.3, 1400.0)	1250.0	±	233.0	1230.0 (1100.0, 1400.0)	1268.4	±	204.1	1270.0 (1134.0, 1400.0)	0.271
Oral conditions													
Dysphagia screening rating													
No dysphagia	97		(21.3)		17		(9.9)		79		(27.8)		<0.001
At risk of dysphagia	175		(38.5)		43		(25.1)		129		(45.4)		
Possibility of dysphagia	183		(40.2)		111		(46.9)		76		(26.8)		
Present teeth	8.3	±	8.9	5.0 (0.0, 16.0)	5.8	±	7.5	3.0 (0.0, 10.25)	9.9	±	9.4	7.0 (0.0, 18.0)	<0.001
Functional teeth	20.1	±	10.2	26.0 (13.0, 28.0)	15.3	±	11.7	16.5 (2.0, 28.0)	23.0	±	7.9	27.5 (20.8, 28.0)	<0.001
Edentulous	155		(34.3)		75		(44.1)		80		(28.4)		0.001
Prosthesis use	243		(53.8)		71		(41.8)		172		(61.0)		<0.001
Medical history													
Pneumonia	32		(7.0)		24		(14.0)		8		(2.8)		<0.001
Stroke	154		(33.9)		53		(31.0)		101		(35.7)		0.357
Diabetes mellitus	66		(14.5)		26		(15.2)		40		(14.1)		0.784
Depression	33		(7.3)		9		(5.3)		24		(8.5)		0.263

Categorical variables are expressed as number (percentage) and were analyzed using the chi-square test. A *p* value of <0.05 was considered statistically significant. Continuous variables were analyzed using the Mann-Whitney U test. A *p* value of <0.05 was considered statistically significant. Q1: first quartile; Q3: third quartile; SD: standard deviation.

**Table 2:** Baseline comparisons between the weight loss group and maintenance group in the regular diet group.

Variable	Regular Diet 2018 (*n* = 284)								
	Weight Loss <5% (*n* = 204)				Weight Loss ≥5% (*n* = 80)				*p* Value
	Mean ± SD			Median, (Q1, Q3)	Mean ± SD			Median (Q1, Q3)	
	*n* (%)				*n* (%)				
Age (years)	85.6	±	7.9	86.0 (80.0, 92.0)	86.7	±	7.8	87.0 (82.5, 92.8)	0.335
Sex (female)	167		(81.9)		64		(80.0)		0.736
Weight (kg)	47.7	±	6.2	47.1 (40.9, 53.4)	49.1	±	8.7	48.1 (42.4, 54.6)	0.214
Barthel Index	44.6	±	25.3	45.0 (25.0, 65.0)	42.6	±	22.2	45.0 (25.0, 60.0)	0.583
Clinical Dementia Rating									
0	8		(3.9)		0		(0.0)		
0.5	30		(14.7)		4		(5.1)		0.058
1	54		(26.5)		25		(31.6)		
2	64		(31.4)		31		(39.2)		
3	48		(23.5)		19		(24.1)		
Total energy intake (kcal/day)	1269.8	±	199.5	1254.5 (1136.8, 1400.0)	1264.7	±	216.8	1302.0 (1100.0, 1400.0)	0.938
Oral conditions									
Dysphagia screening rating									
No dysphagia	58		(28.4)		21		(26.3)		0.564
At risk of dysphagia	95		(46.6)		34		(42.5)		
Possibility of dysphagia	51		(25.0)		25		(31.3)		
Present teeth	9.6	±	9.3	7.0 (0.0, 18.0)	10.5	±	9.6	7.5 (0.0, 20.0)	0.497
Functional teeth	23.2	±	7.7	28.0 (21.8, 28.0)	22.3	±	8.4	27.0 (20.0, 28.0)	0.274
Edentulous	58		(28.7)		22		(27.5)		0.884
Prosthesis use	128		(63.4)		44		(55.0)		0.223
Medical History									
Pneumonia	4		(2.0)		4		(5.0)		0.228
Stroke	72		(35.5)		29		(36.3)		0.891
Diabetes mellitus	30		(14.8)		10		(12.5)		0.707
Depression	19		(9.49)		5		(6.3)		0.484

Categorical variables are expressed as number (percentage) and were analyzed using the chi-square test. A *p* value of <0.05 was considered statistically significant. Continuous variables were analyzed using the Mann-Whitney U test. A *p* value of <0.05 was considered statistically significant. Q1, first quartile; Q3, third quartile; SD, standard deviation.

**Table 3:** Comparison of the weight loss and weight maintenance group according to the changes in the regular diet group for 1 year.

Variable (Change/Year)	Overall (*n* = 284)				Weight Loss <5% (*n* = 204)				Weight Loss ≥5% (*n* = 80)				*p* Value
	Mean ± SD			Median (Q1, Q3)	Mean ± SD			Median (Q1, Q3)	Mean ± SD			Median (Q1, Q3)	
	*n* (%)				*n* (%)				*n* (%)				
Barthel Index	−5.2	±	15.8	−5.0 (−15.0, 5.0)	−3.3	±	15.5	0.0 (−10.0, 5.0)	−10.0	±	15.6	−5.0 (−20.0, 0.0)	0.003
Clinical Dementia Rating deterioration	+86		(+30.3)		+56		(+27.5)		+30		(+37.5)		0.114
Total energy intake (kcal/day)	+13.1	±	203.1	0.0 (−100.0, 100.0)	+28.6	±	183.8	0.0 (−75.0, 107.0)	−23.5	±	240.2	0.0 (−187.8, 79.0)	0.095
Dysphagia screening rating deterioration	+91		(+32.0)		+61		(+29.9)		+30		(+37.5)		0.258
Functional teeth	−1.1	±	5.8	0.0 (0.0, 0.0)	−1.0	±	5.9	0.0 (0.0, 0.0)	−1.4	±	5.4	0.0 (−2.0, 0.0)	0.151
Food form (Dysphagia diet)	+55		(+19.4)		+27		(+13.2)		+28		(+35.0)		<0.001

Categorical variables are shown as number (percentage) and were analyzed using the chi-square test. A *p* value of <0.05 was considered statistically significant. Continuous variables were analyzed using the Mann-Whitney U test. A *p* value of <0.05 was considered statistically significant. Q1, first quartile; Q3, third quartile; SD, standard deviation.

**Table 4:** Logistic regression analysis of the presence or absence of ≥5% weight loss in 1 year.

	OR	95% CI			*p* Value
Age (years)	1.03	0.98	–	1.07	0.232
Sex (female)	1.21	0.49	–	2.95	0.683
Weight (kg) Baseline	1.04	1.00	–	1.08	0.031
Barthel Index change	0.97	0.95	–	0.99	0.009
Clinical Dementia Rating deterioration	1.07	0.54	–	2.10	0.852
Total energy intake change (kcal/day)	1.00	1.00		1.00	0.407
Food form change (to dysphagia diet)	3.02	1.41	–	6.46	0.004
Dysphagia screening rating deterioration	1.02	0.52	–	1.97	0.962
Functional teeth change	0.99	0.94	–	1.04	0.680
Pneumonia	2.42	0.46	–	12.83	0.299
Stroke	1.13	0.59	–	2.15	0.711
Diabetes mellitus	0.78	0.30	–	2.04	0.612
Depression	1.11	0.34	–	3.64	0.863

CI = Confidence interval, logistic regression analysis, Dependent variable: Weight loss 5%. Independent variables: age, sex, weight at baseline, medical history, BI, CDR, total energy intake, dysphagia screening rating, functional teeth, food form change.

## Data Availability

The data presented in this study are available on request from the corresponding author. The data are not publicly available due to ethico-legal restrictions imposed by the Ethics Committee at the Japanese Society of Gerodontology.

## References

[1] Wirth R., Streicher M., Smoliner C., Kolb C., Hiesmayr M., Thiem U., Sieber C.C., Volkert D. (2016). The impact of weight loss and low BMI on mortality of nursing home residents—Results from the nutritionDay in nursing homes. Clin. Nutr..

[2] He W., Goodkind D., Kowal P. (2016). An Aging World 2015.

[3] Arai H., Ouchi Y., Yokode M., Ito H., Uematsu H., Eto F., Oshima S., Ota K., Saito Y., Sasaki H. (2012). Toward the realization of a better aged society: Messages from gerontology and geriatrics. Geriatr. Gerontol. Int..

[4] Health and Welfare Bureau for the Elderly Ministry of Health, Labour and Welfare Long-Term Care Insurance System of Japan. https://www.mhlw.go.jp/english/policy/care-welfare/care-welfare-elderly/dl/ltcisj_e.pdf.

[5] Flynn E., Smith C.H., Walsh C.D., Walshe M. (2018). Modifying the consistency of food and fluids for swallowing difficulties in dementia. Cochrane Database Syst. Rev..

[6] Edahiro A., Hirano H., Yamada R., Chiba Y., Watanabe Y. (2013). Comparative study of eating behavior in elderly patients with Alzheimer’s disease and vascular dementia: A first report—Comparison of disturbed eating behavior. Nihon Ronen Igakkai Zasshi Jpn. J. Geriatr..

[7] Kuzuya M. (2003). Nutritional assessment and nutritional management for the elderly. Nippon Ronen Igakkai Zasshi. Jpn. J. Geriatr..

[8] Robbins J., Gensler G., Hind J., Logemann J.A., Lindblad A.S., Brandt D., Baum H., Lilienfeld D., Kosek S., Lundy D. (2008). Comparison of 2 interventions for liquid aspiration on pneumonia incidence: A randomized trial. Ann. Intern. Med..

[9] Painter V., Le Couteur D., Waite L. (2017). Texture-modified food and fluids in dementia and residential aged care facilities. Clin. Interv. Aging.

[10] Keller H., Chambers L., Niezgoda H., Duizer L. (2012). Issues associated with the use of modified texture foods. J. Nutr. Health Aging.

[11] Germain I., Dufresne T., Gray-Donald K. (2006). A novel dysphagia diet improves the nutrient intake of institutionalized elders. J. Am. Diet Assoc..

[12] Dietitians Association of Australia, The Speech Pathology Association of Australia Limited (2007). Texture-modified foods and thickened fluids as used for individuals with dysphagia: Australian standardised labels and definitions. Nutr. Diet.

[13] Mahoney F.I., Barthel D.W. (1965). Functional Evaluation: The Barthel Index. Md. State Med. J..

[14] Morris J.C. (1993). The Clinical Dementia Rating (CDR): Current version and scoring rules. Neurology.

[15] Matsuo K., Fujishima I. (2020). Textural changes by mastication and proper food texture for patients with oropharyngeal dysphagia. Nutrients.

[16] Fujitani J., Uyama R., Oogosi H. (2013). Japanese Dysphagia Diet 2013 by the JSDR dysphagia diet committee (JDD2013). Dysphagia Rehabil..

[17] Ohkuma R., Fjishima I., Kojima C., Hojo K., Takedhara I., Motohashi Y. (2002). Development of a questionnaire to screen dysphagia. Jpn. J. Dysphagia Rehabil..

[18] Ohkuma R., Fjishima I. (2012). Development of a The Relationship between the Seirei Questionnaire of Swallowing and 30 ml Water Swallowing Test. Jpn. J. Dysphagia Rehabil..

[19] Kawashima K., Motohashi Y., Fujishima I. (2004). Prevalence of dysphagia among community-dwelling elderly individuals as estimated using a questionnaire for dysphagia screening. Dysphagia.

[20] Wallace J.I., Schwartz R.S., LaCroix A.Z., Uhlmann R.F., Pearlman R.A. (1995). Involuntary weight loss in older outpatients: Incidence and clinical significance. J. Am. Geriatr. Soc..

[21] De Stefani F.D.C., Pietraroia P.S., Fernandes-Silva M.M., Faria-Neto J., Baena C.P. (2018). Observational evidence for unintentional weight loss in all-cause mortality and major cardiovascular events: A systematic review and meta-analysis. Sci. Rep..

[22] Gazewood J.D., Mehr D.R. (1998). Diagnosis and management of weight loss in the elderly. J. Fam. Pract..

[23] Nishida Y., Tanaka S., Nakae S., Yamada Y., Shirato H., Hirano H., Sasaki S., Katsukawa F. (2020). Energy Gap between Doubly Labeled Water-Based Energy Expenditure and Calculated Energy Intake from Recipes and Plate Waste, and Subsequent Weight Changes in Elderly Residents in Japanese Long-Term Care Facilities: CLEVER Study. Nutrients.

[24] Valentini L., Schindler K., Schlaffer R., Bucher H., Mouhieddine M., Steininger K., Tripamer J., Handschuh M., Schuh C., Volkert D. (2009). The first nutritionDay in nursing homes: Participation may improve malnutrition awareness. Clin. Nutr..

[25] Watanabe I. (1998). Masticatory function and life style in aged. Nihon Ronen Igakkai Zasshi Jpn. J. Geriatr..

[26] Saka B., Kaya O., Ozturk G.B., Erten N., Karan M.A. (2010). Malnutrition in the elderly and its relationship with other geriatric syndromes. Clin. Nutr..

[27] McKendry J., Currier B.S., Lim C., McLeod J.C., Thomas A.C.Q., Phillips S.M. (2020). Nutritional supplements to support resistance exercise in countering the sarcopenia of aging. Nutrients.

[28] Azzolino D., Passarelli P.C., De Angelis P., Piccirillo G.B., D’Addona A., Cesari M. (2019). Poor Oral Health as a Determinant of Malnutrition and Sarcopenia. Nutrients.

[29] Motokawa K., Yasuda J., Mikami Y., Edahiro A., Morishita S., Shirobe M., Ohara Y., Nohara K., Hirano H., Watanabe Y. (2020). The Mini Nutritional Assessment-Short Form as a predictor of nursing home mortality in Japan: A 30-month longitudinal study. Arch. Gerontol. Geriatr..

[30] Park M., Song J.A., Lee M., Jeong H., Lim S., Lee H., Kim C.G., Kim J.S., Kim K.S., Lee Y.W. (2018). National study of the nutritional status of Korean older adults with dementia who are living in long-term care settings. Jpn. J. Nurs. Sci..

[31] Chiesi F., Grazzini M., Innocenti M., Giammarco B., Simoncini E., Garamella G., Zanobini P., Perra C., Baggiani L., Lorini C. (2019). Older people living in nursing homes: An oral health screening survey in Florence, Italy. Int. J. Environ. Res. Public Health.

